# Analyses of Saliva Metabolome Reveal Patterns of Metabolites That Differentiate SARS-CoV-2 Infection and COVID-19 Disease Severity

**DOI:** 10.3390/metabo15030192

**Published:** 2025-03-11

**Authors:** Violeta Larios-Serrato, Natalia Vázquez-Manjarrez, Osbaldo Resendis-Antonio, Nora Rios-Sarabia, Beatriz Meza, Oliver Fiehn, Javier Torres

**Affiliations:** 1 Departamento de Bioquímica, Escuela Nacional de Ciencias Biológicas, Instituto Politécnico Nacional, Mexico City 11340, Mexico; viosdatafactory@gmail.com; 2 Unidad de Metabolómica, Departamento Fisiologia de la Nutricion, Instituto de Ciencias Médicas y Nutrición Salvador Zubirán, Mexico City 14080, Mexico; natalia.vazquezm@incmnsz.mx; 3 Human Systems Biology Laboratory, Instituto Nacional de Medicina Genómica, Centro de Ciencias de la Complejidad & Coordinación de la Investigación Científica-Red de Apoyo a la Investigación, Universidad Nacional Autonoma de Mexico, Mexico City 04510, Mexico; oresendis@inmegen.gob.mx; 4 Unidad de Investigación Médica en Enfermedades Infecciosas, UMAE Pediatría, Centro Médico Nacional SXXI, Instituto Mexicano del Seguro Social, Mexico City 06920, Mexico; noraisela19@gmail.com; 5 Departamento Académico de Ciencia Animal y Conservación del Hábitat, Departamento Académico de Ingenierías en Pesquerías, Universidad Autónoma de Baja California Sur, La Paz 023080, Mexico; mezamarquezbeatriz@gmail.com; 6 Centro de Investigaciones Biológicas del Noroeste SC, La Paz 23205, Mexico; 7 NIH West Coast Metabolomics Center, UC Davis Genome Center, Davis, CA 95616, USA; ofiehn@ucdavis.edu

**Keywords:** metabolome, COVID-19, severity, patient

## Abstract

Background: The metabolome of COVID-19 patients has been studied sparsely, with most research focusing on a limited number of plasma metabolites or small cohorts. This is the first study to test saliva metabolites in COVID-19 patients in a comprehensive way, revealing patterns significantly linked to disease and severity, highlighting saliva’s potential as a non-invasive tool for pathogenesis or diagnostic studies. Methods: We included 30 asymptomatic subjects with no prior COVID-19 infection or vaccination, 102 patients with mild SARS-CoV-2 infection, and 61 hospitalized patients with confirmed SARS-CoV-2 status. Saliva samples were analyzed using hydrophilic interaction liquid chromatography–mass spectrometry (HILIC-MS/MS) in positive and negative ionization modes. Results: Significant differences in metabolites were identified in COVID-19 patients, with distinct patterns associated with disease severity. Dipeptides such as Val-Glu and Met-Gln were highly elevated in moderate cases, suggesting specific protease activity related to SARS-CoV-2. Acetylated amino acids like N-acetylserine and N-acetylhistidine increased in severe cases. Bacterial metabolites, including muramic acid and indole-3-carboxaldehyde, were higher in mild–moderate cases, indicating that oral microbiota differs according to disease severity. In severe cases, polyamines and organ-damage-related metabolites, such as N-acetylspermine and 3-methylcytidine, were significantly increased. Interestingly, most metabolites that were reduced in moderate cases were elevated in severe cases. Conclusions: Saliva metabolomics offers insightful information that is potentially useful in studying COVID-19 severity and for diagnosis.

## 1. Introduction

SARS-CoV-2 continues to circulate and evolve in human populations, with approximately 161,000 new cases reported globally during the 28-day period in January 2025, despite extensive vaccination efforts. With over 7 million deaths, it is clear that early SARS-CoV-2 episodes were fierce, with a high mortality rate [1]. The human innate immunological response was unprepared for SARS-CoV-2, leading to a dysregulated and hyperinflammatory response that contributed significantly to severe and fatal cases [2]. Currently, a human/SARS-CoV-2 co-adaption process and vaccination programs have led to a reduced mortality rate and a more trained innate (monocyte/macrophage trained due to prior infections) or adaptive immune response (due to vaccination and prior infections). A modulation of the inflammatory response is required to fight back the infection and reduce the damage to the patient [3].

Understanding the initial interactions between SARS-CoV-2 and naïve human populations provides critical insights into the immune response to novel zoonotic viruses, a risk that remains ever-present [4]. A key element in this scenario is the microenvironment at the site of infection, which is intricately influenced by the virus, epithelial cells, immune responses, cytokines, and antiviral molecules, which collectively contribute to the complex etiology of the disease. The upper respiratory tract harbors a rich microbial community in humans, second in diversity only after the gut microbiota [5]. This microbial community has the capacity to modulate infectivity, transmission, and even the severity of viral infections through three mechanisms: (i) priming the immune system through certain bacterial species, thereby enhancing antiviral defenses; (ii) restricting viral entry via commensal bacteria that compete for host receptors or produce antimicrobial substances that indirectly affect viral infectivity; or (iii) modulating the inflammatory response, which may either exacerbate or mitigate viral disease severity [6].

The microbiota represents a key element in defining the composition of the metabolome in the respiratory tract microenvironment, directly producing specific metabolites or impacting the metabolic activity of the epithelial and immune cells in this niche. However, there are limited reports that study the metabolome of the respiratory tract in COVID-19 patients. Saliva has emerged as a promising matrix for exploration. We recently reported significant changes in the microbiota of the saliva of patients with SARS-CoV-2 infection [7]. Our findings demonstrated that the diversity, composition, and networking of the microbial communities differed according to the severity of the disease. The metabolic activity of these communities was inferred with Picrust2 software, and significant differences were predicted, particularly in the metabolism of amino acids, fatty acids, and antibiotics. A comprehensive characterization of the metabolome within the oral cavity is expected to yield insights into the establishment of the viral infection “culture medium” and provide crucial information to elucidate the pathogenesis and severity of the disease [8]. Saliva is a non-invasive, readily accessible biofluid that can be collected without the need for specialized personnel. However, it remains underutilized in metabolomic research for health and disease. As a biofluid, saliva reflects both systemic and local metabolites, with the potential to inform about metabolic signatures associated with diverse diseases, particularly in respiratory tract infections, which makes it a valuable matrix for diagnostic and prognostic purposes [9].

In this work, we aimed to thoroughly analyze the metabolome in saliva samples from patients who acquired SARS-CoV-2 infection during the early phase of the pandemic, prior to the development of vaccines and without previous infection. In addition, we aimed to elucidate possible differences between cases with diverse disease severity. The results showed that SARS-CoV-2 infection was significantly associated with distinct metabolomic profiles.

## 2. Materials and Methods

### 2.1. Study Population

Patients were recruited from June 2020 to January 2021, before any vaccination program was in place and during a period characterized by uncertain epidemiologic and clinical scenarios with strict containment measures, including evolving treatment protocols, a limited understanding of COVID-19 progression, and the absence of widely available vaccines. This study was approved in May 2020 by the IRB (Institutional Review Board), the ethics committee, and the National Research Commission of the Instituto Mexicano del Seguro Social, Mexico (registry number R-2020-785-053). During this time, there was a total absence of treatment protocols and the natural history of the disease was unknown. Therefore, patient recruitment had to be conducted by the attending health personnel of the Hospital General de Zona con Unidad Medicina Familiar No. 8 in Mexico City. A group of mild cases included ambulatory patients with mild respiratory symptoms (fever, cough, headache, odynophagia, myalgias) presenting for COVID-19 diagnosis, and recruited before any prescribed treatment (AP group). Patients who, during follow-up, required hospitalization because they developed severe symptoms were excluded from this group and included in the hospitalized group. The group of severe patients included cases that required hospitalization because of severe symptoms (HP group), with oxygen saturation below 92% and the presence of risk comorbidities including hypertension, diabetes, morbid obesity, immunocompromised, cardiovascular or neurological diseases, chronic renal failure, tuberculosis, or neoplasia. Patients were usually hospitalized within the first seven days after symptoms started and followed until discharged because of recovery, improvement, or death. Patients with suspicious symptoms before a confirmed diagnosis participated in this study. After testing for SARS-CoV-2 (COBAS 6800, Merck Mexico, Mexico City, Mexico), they were classified as positive or negative for the infection. Individuals without symptoms and no previous COVID-19 infection were included in the asymptomatic case (AC) group. Smokers and those with any chronic disease were excluded. Patients who consented were asked to sign an informed consent letter. A summary of the clinical groups studied is shown in Table 1.

### 2.2. Collection and Processing of Saliva Samples for Untargeted Metabolomics

Patients were asked to thoroughly wash their mouths with 10 mL of saline solution (0.85% NaCl) and spit back into a 50 mL plastic tube. Samples were immediately transported to a central laboratory for SARS-CoV-2 diagnosis. On arrival, samples were inactivated by heating at 65 °C for 30 min. After the diagnosis, samples were sent to the UC Davis West Coast Metabolomics Center, Fiehn Lab, following international safety regulations. Samples were frozen at −70 °C until studied. The sample preparation procedure was performed according to standard protocols for untargeted metabolomics. After extraction, the samples were analyzed using hydrophilic interaction liquid chromatography–mass spectrometry (HILIC-MS/MS) in positive and negative ionization modes to generate the metabolomic data. Quality control was ensured through the use of 40 deuterated internal standards, CUDA, and the tripeptide Val-Tyr-Val. These standards were employed to evaluate peak shapes, and raw peak intensities during the runs, and to normalize retention times.

### 2.3. Data Preprocessing and Quality Control

Samples were processed on a Sciex TripleTOF 6600 high-resolution mass spectrometer (SCIEX, Redwood City, CA, USA)**,** using a Waters BEH Amide 2.1 × 50 mm column with 1.7 um particles and an acetonitrile/water gradient [10]. Raw LC-MS files were processed by MS-DIAL 4.0 software [11]. A total of 3452 features were obtained in positive (2218) and negative (1234) modes. For quality control, data for blanks and pool quality controls were acquired after every 10 samples. Metabolites were removed if they had signal/noise ratios (s/n) smaller than 3:1 when comparing samples over blank controls or removed if s/n < 10 for unknown compounds. MS-FLO software v1.8 was used to integrate adducts and remove duplicates. In order to normalize, pool quality controls were used for signal drifts using Systematic Error Removal with Random Forest (SERFF) [12]. Compounds were annotated by matching accurate mass and MS/MS data against NIST17 and MassBank of North America libraries. Principal Component Analysis (PCA) was applied for quality control to assess the overall sample distribution and detect any outliers or batch effects (Supplementary Figure S1).

### 2.4. Statistical Analysis, Variable Selection, and Performance Assessment

The statistical analysis began with the selection of main ions pertinent to the biological question by Partial Least Squares Discriminant Analysis (PLS-DA) using MATLAB R2023b and PLS toolbox 9.1. Only annotated ions were retained for further analysis, resulting in a size 308 × 905 data matrix. The data matrix was divided into a calibration set and a test set, with 10% of the samples assigned to the test set. PLS-DA was performed on the data matrix to discriminate between the groups, using Pareto-scaled data with 100 iterations and leave-one-out cross-validation for the inner loop to optimize the model’s parameters. The groups analyzed included AC vs. AP, AC vs. HP, and AP vs. HP (see Table 1). The selected features from PLS-DA were then applied to the test set to evaluate their discriminative performance. Performance metrics included the Area Under the Receiver Operating Characteristic Curve (AUROC) and the misclassification error. Variable selection was conducted using the selectivity ratio and Variable Importance in Projection (VIP) values. Features identified as highly discriminant in at least 80% of the models were considered relevant to the biological question. Differences in metabolites between experimental groups were further analyzed to identify compounds that significantly distinguished each group, using R 4.4.0 packages and Rstudio 2024. One-way Analysis of Variance (ANOVA) was used to explore the data with multigroups, and the False Discovery Rate (FDR) was calculated to identify important compounds among all comparisons (*p* < 0.05). Violin plots were built with the ggplot2 v3.5.1 package [13]. The geometric mean for each metabolite across all samples was calculated using DESeq2 v1.44.0 [14] and normalized using the total sum scale (TSS). To analyze the differences in the abundance of metabolites between groups, the fold change (FC) was calculated, and *p*-values were obtained using the Wald test. These values were corrected for multiple tests using the Benjamini and Hochberg method by default. Volcano plots were created with EnhancedVolcano v1.12.0.

### 2.5. Data Availability

The database of the metabolome is described in Supplementary Table S1, showing information on the assays, intensities, and internal standards.

## 3. Results

### 3.1. The Composition of Saliva Metabolites Clearly Distinguished the Clinical Groups

**Figure 1:**
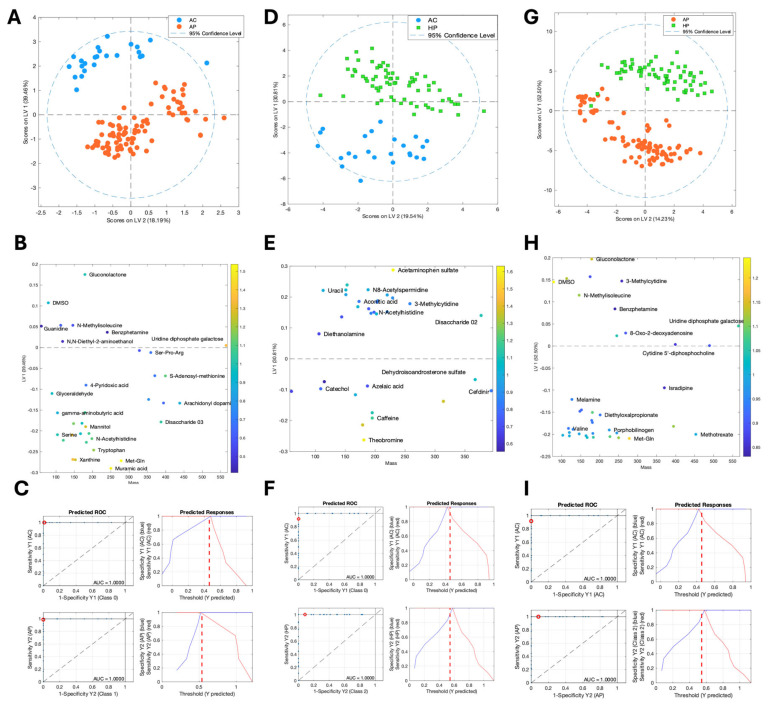
PLS-DA score plots, metabolite selection, and predictive performance across clinical groups. PLS-DA results for AC vs. AP (**A**–**C**), AC vs. HP (**D**–**F**), and AP vs. HP (**G**–**I**). Panels (**A**,**D**,**G**) show PLS-DA score plots illustrating the metabolic separation between clinical groups: asymptomatic cases (ACs), moderate ambulatory patients (APs), and severe hospitalized cases (HPs). Panels (**B**,**E**,**H**) display the corresponding loadings plots, where metabolites are highlighted based on their Variable Importance in Projection (VIP) values. Panels (**C**,**F**,**I**) present predictive performance metrics, including AUROCs (left) and predicted response plots (right), where the red dashed line represents the classification threshold. ACs, asymptomatic cases; APs, ambulatory patients; HPs, hospitalized patients.

To identify the metabolites that contributed the most to the observed group separation, we conducted pairwise PLS-DA comparisons (Figure 1). The results identified 35 metabolites distinguishing AC (asymptomatic cases) from AP (moderate ambulatory patients), 33 differentiating AC from HP (severe hospitalized cases), and 37 separating AP from HP. A literature review indicated that these metabolites originated from human, environmental, or microbial sources (Table 2).

Furthermore, we assessed the predictive ability of these metabolic profiles using receiver operating characteristic (ROC) analysis. The area under the curve (AUC) values demonstrated excellent classification performance for all pairwise comparisons (Figure 1). Supplementary Table S2 provides a detailed breakdown of metabolite intensity differences across the groups.

### 3.2. Amino Acid Metabolites Were Strongly Associated with Moderate COVID Cases and Bacterial Metabolites Were Srongly Associated with Severe Cases

**Figure 2:**
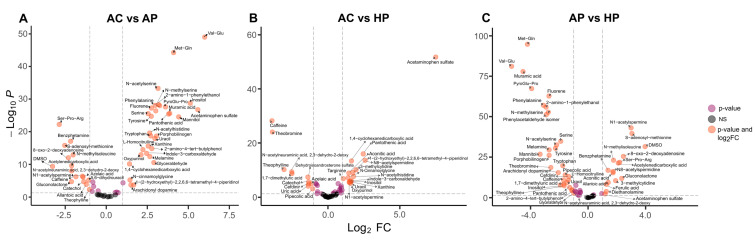
Pairwise differential abundance analysis of metabolites among the three clinical groups. The volcano plot shows metabolite fold changes (FCs) on the *x*-axis and the False Discovery Rate (FDR) on the *y*-axis. Dashed lines indicate the threshold, FC ≥ 1.0 (either positive or negative), and FDR > −log (0.05). Metabolites with significant abundance are depicted as orange dots. (**a**) Comparison between the asymptomatic control group (AC) and the ambulatory SARS-CoV-2-positive group (AP); (**b**) comparison between the asymptomatic control group (AC) and the hospitalized SARS-CoV-2-positive group (HP); (**c**) comparison between ambulatory patients (AP) and the hospitalized SARS-CoV-2-positive group (HP).

**Figure 3:**
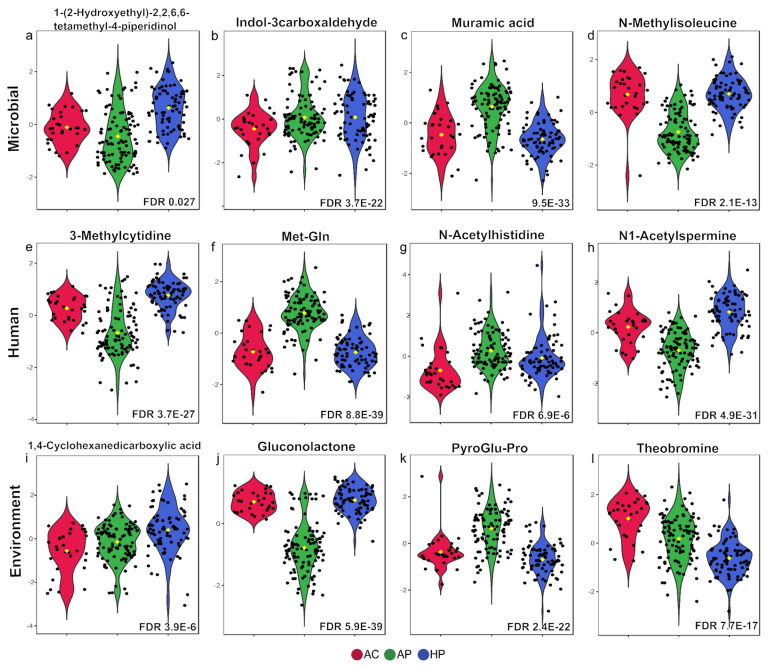
(**a**–**l**) Violin plots of metabolites with differences among the AC, AP, and HP groups, according to their source. A selection of metabolites for three categories (microbial, human, environment) is shown. False Discovery Rate (FDR) values (<0.05) with one-way ANOVA are indicated.

The presence of SARS-CoV-2 infection was associated with markedly significant differences in metabolites in saliva, particularly in moderate cases when compared with the asymptomatic group. The contribution of each metabolite to the differentiation of these groups was determined using a volcano plot (Figure 2). When comparing AP with the AC group, a significantly higher concentration of metabolites was observed. Notably, dipeptides, such as Val-Glu and Met-Gln, showed highly significant values in AP (*p*-value > 10^−40^) (Figure 3). Additionally, amino acids and their derivatives, including serine, phenylalanine, tyrosine, N-acetylserine, and N-methyl serine, presented higher abundance in AP subjects (*p*-values > 10^−20^). In contrast, in the AC group, Ser-Pro-Arg (*p*-value < 10^−20^), S-adenosyl-methionine, and N-methylisoleucine (*p*-values around 10^−10^) were significantly higher.

The metabolome of HPs presented a different pattern when compared with ACs (Figure 2b), with the concentration of three bacteria-derived metabolites, pantothenic acid, 1-(2-Hydroxyethyl)-2,2,6,6-tetramethyl-4-piperidinol, and indole-3-carboxaldehyde, being significantly higher. Also, human-derived N8-acetylspermidine and 3-methylcytidine concentrations were significantly higher, whereas amino acids or their derivatives were almost absent (as opposed to results in the AP group). In contrast, the saliva of AC individuals was significantly enriched with human-derived 1,7 dimethyl uric acid, N-acetylneuraminic acid, and dehydroisoandrosterone sulfate.

We next explored the differences between the two SARS-CoV-2-infected groups, AP and HP (Figure 2c), and observed that N1-acetylspermidine, 8-oxo-2-deoxyadenosine, S-adenosyl-methionine, and dimethyl-sulfoxide (DMSO) were significantly enriched in the HP group (*p*-value 10^−25^). N-methyl isoleucine, Ser-Pro-Arg, acetylene dicarboxylic acid, and N8-acetylspermidine were also significantly more abundant in HPs. In contrast, amino acids and their derivatives, Met-Gln and Val-Glu (*p*-values > 10^−80^); phenylalanine, pyroGlu-Pro, and N-methylserine (*p*-values > 10^−50^); and serine, N-acetylserine, and tyrosine (*p*-values > 10^−25^) were extremely higher in the AP group. Furthermore, muramic acid and 2-amino-1-phenylethanol were two bacterial products highly enriched in AP (*p*-value 10^−50^).

As shown in Figure 3, the concentration of certain metabolites exhibited significantly different concentrations between the groups. Metabolites are presented according to their origin, whether microbial, human, or environmental.

### 3.3. Some Metabolites Distinguish Deceased from Severe-Hospitalized Patients

In the group of hospitalized patients, there were 17 deceased cases (DHP), and we searched for any difference between these 17 and the remaining 44 hospitalized patients (HPs). S-adenosyl-methionine, N-acetylhistidine, porphobilinogen, tyrosine, and bacteria-derived muramic acid were significantly increased in DHP compared to those in the HP group (Supplementary Figure S2). In contrast, only 2-amino-4 tertbutylphenol was significantly enriched in the HP group.

## 4. Discussion

While several metabolomic studies in COVID-19 patients have been published, most of them reported metabolites in plasma with a limited number of samples [15,16]. The few studies conducted in saliva included a smaller number of patients and a more limited search for metabolites [17,18,19]. The present work is a comprehensive analysis of metabolites present in the saliva of patients with COVID-19, showing significant differences in the composition and concentration of metabolites in patients infected with SARS-CoV-2 and specific metabolite patterns associated with disease severity. We discuss our work in the context of both the utility of our findings to better understand pathogenic mechanisms, but also in the search for metabolites as potential candidates for diagnosis or disease severity.

The presence of the dipeptides Val-Glu and Met-Gln in the saliva of patients with moderate COVID disease was significantly higher than in asymptomatic uninfected individuals. We did not find any report of these peptides in saliva, but saliva is known to contain several proteolytic enzymes [20] and peptides could be the products of these enzymes. The marked abundance of only these two dipeptides would suggest that in mild–moderate COVID episodes, SARS-CoV-2 infection induces the activity of specific proteases. Previous studies have suggested that saliva contains a mixture of enzymes that are resistant to protease inhibitors but with a tightly controlled activity that has been associated with health and disease [21,22]. Some dipeptides may present cell-signaling effects [23] or regulatory functions important for health [24]. Val-Glu and Met-Gln may be useful for the diagnosis of the infection.

Phenylalanine, serine, tyrosine, and tryptophan also increased in cases of mild–moderate disease, and may also result from proteolytic activity, but could also be of microbial origin. Recent studies have shown that tryptophan catabolites produced by microbial members may have relevant roles in health and disease, and they may stimulate the immune system, epithelial barrier, and even gut hormone secretion [25]. In contrast to our results, an exploratory study by Frampas et al. reported a decrease in phenylalanine and tyrosine in the saliva of COVID-19-infected patients [26]. Patients in our study were recruited during the first wave of the pandemic, before vaccination, and this might be an important difference from other reports. In any case, it is important to explore which proteases are activated during SARS-CoV-2 infection and the role their products may have in the pathogenesis of COVID-19.

Two acetylated amino acids that are strongly associated with mild–moderate COVID-19, N-acetylserine and N-acetylhistidine, have also been reported to be elevated in the sera of patients with chronic kidney disease and were suggested as markers of tubular renal function [27,28]. In addition, N-acetylhistidine was found to increase during ischemia in pigs with induced myocardial infarction [29] but was reported to be significantly decreased in the brains of Alzheimer‘s disease patients [30]. The N-terminal acetylation of amino acids has been suggested as part of the response to stress and has been associated with cancer and other developmental disorders [31]. In previous studies, acetylated amino acids have been detected mostly in sera. Our study shows for the first time that they can also be detected in saliva and suggests that saliva N-acetylserine and N-acetylhistidine might be useful as markers for patients at risk of developing more severe COVID-19 disease.

Muramic acid, a component of the bacterial cell wall, was significantly increased in mild–moderate cases, suggesting that there might be an overgrowth of bacteria as a response to SARS-CoV-2 infection. Indole-3-carboxaldehyde, a bacterial metabolite of tryptophan, also increased in mild–moderate cases. This metabolite is synthesized particularly by *Lactobacillus* spp. and is known to stimulate IL-22 production and increase the immune reactivity of mucosa [32]. The increase in these two metabolites attests to relevant changes in the oral microbiota of patients infected with SARS-CoV-2.

Ser-Pro-Arg was significantly reduced in mild–moderate patients, which is probably a product of proteolytic activity. We did not find reports of this peptide in saliva or other human fluids, although its sequence was present in at least five repeats in the histone H1, where they might be important for the structure of the protein [33].

S-adenosyl-methionine (SAM), a key metabolite in methylation reactions, was significantly reduced in mild–moderate patients, which suggests that it might be consumed during infection. SAM is produced in the liver and has been associated with liver disease, including steatosis, hepatocarcinoma, or hepatomegaly, and might be a sensitive marker for liver damage, one of the multiple targets of SARS-CoV-2 [34]. It is interesting to note that the administration of SAM to patients with HCV inhibits the expression of the virus and improves the activity of peginterferon [34]. Also, N-methylisoleucine was reduced in mild–moderate patients, and this compound has been found to be upregulated in the plasma of patients with glioblastoma [35]. It is tempting to suggest that Ser-Pro-Arg, SAM, and N-methylisoleucine are amino acid metabolites that are significantly depleted during acute SARS-CoV-2 infection to prevent severe disease.

The bacterial metabolites pantothenic acid and indole-3-carboxaldehyde significantly increased in the hospitalized patients (Figure 2b). Pantothenic acid is a precursor in the synthesis of CoA and studies have revealed that many Bacteroidetes and Proteobacteria produce it [36]. Indole is produced by many bacteria and multiple physiological functions have been described, including plasmid stability, drug resistance, or biofilm formation [37]. The observed increase in these two metabolites in severe cases as compared to asymptomatic individuals suggests differences in bacterial composition.

Polyamines are essential for cell survival, participate in multiple processes like metabolism or the regulation of replication, and have been associated with multiple diseases [38]. N8-acetylspermidine is a polyamine produced in episodes of cardiac ischemia, cancer, or even infections, usually detected in plasma [39]. A study in patients with gastric cancer reported that increased plasma levels of acetylated polyamines correlated with severe cases [16]. Here, we found increased levels in the saliva of patients with severe COVID-19 infection, probably as an alert sign for the extensive damage to multiple organs.

1-(2-Hydroxyethyl)-2,2,6,6-tetramethyl-4-piperidinol is not a human metabolite but is found in the blood of people exposed to a possible source such as additive stabilizer in the synthesis of plastics or light stabilizers. This compound was increased in the serum of rats exposed to stress, and authors suggested that it may have a role in the gut–brain axis dialogue [40]. In addition, 1-4-cyclohexanedicarboxilic acid is a polyvinyl chloride used as a flavor in food, beverages, and medical applications [41] that was also increased in severe cases. The increased level of these two metabolites in the saliva of hospitalized patients may suggest that previous exposure to these compounds represents a risk of developing severe COVID-19 disease.

3-methylcytidine increased in severe-hospitalized patients. It is a key regulator of transcription, responsible for fine-tuning the gene expression in response to changing conditions, and is considered an epitranscriptome marker [42]. Probably in severe COVID-19, there is a demand for 3-methylcytidine because of a need for increased transcription activity, particularly requiring tRNA m3-C32 modifications.

It was intriguing to note the striking differences in metabolites between moderate and severe cases. Several metabolites that significantly decreased in moderate patients (8-oxo-2-deoxyadenosine, S-adenosyl-methionine, N-methylisoleucine, Ser-Pro-Arg, acetylenedicarboxylic acid, and gluconolactone) showed higher values in the severe cases. Furthermore, the strong dipeptide and amino acid response observed in mild–moderate cases was absent in the severe cases. It is tempting to speculate that the stronger metabolic changes observed in moderate cases represent a robust response that prevents patients from developing severe disease. In contrast, in severe patients, the strong increase was in alarm metabolites like N-acetylspermine, S-adenosyl-methionine, and 8-oxo-2-deoxyadenosine, already described as markers of other severe diseases.

Finally, it is interesting to note that when comparing the deceased cases with the severe patients, the most significant difference was a high increase in N-acetylhistidine in fatal cases, a metabolite that has been associated with kidney failure [28] but also myocardial ischemia [29] and even Alzheimer’s disease [30]. Porphobilinogen was also increased in deceased cases and has been suggested as a marker for hepatic porphyrias and kidney disease [43]. Thus, the detection of N-acetylhistidine and porphobilinogen in saliva may be markers of kidney, heart, or brain damage in severe COVID-19 cases with an increased risk of a fatal outcome.

A limitation of this study was that patients were recruited during the first wave of SARS-CoV-2 infection, when the universal measure was strict restrictions on human contact, and when there was no known treatment to limit the infection or to counteract symptoms and damage to organs. Under these circumstances, it was not possible to control the selection of patients or the correct sampling, and the out-of-proportion demand for medical attention impeded the use of questionnaires to uniformly record clinical and epidemiological information. As a result, several drugs, antimicrobials, food remains, or even compounds from herbal self-medication were found in the saliva samples. Still, highly significant differences were found between groups in this study performed under real-life worldwide crises.

One of the key challenges in analyzing metabolomic differences among groups with different severities is the influence of external factors, including medical interventions. In our study, patients were recruited during the first wave of COVID-19, when there was no standardized therapy. Hospitalized patients likely received supportive care, including oxygen therapy, corticosteroids, antivirals, and other medications that may have influenced their metabolic profiles. For instance, corticosteroids have been reported to modulate amino acid metabolism and inflammatory responses, potentially affecting the levels of metabolites such as S-adenosyl-methionine or polyamines, which were elevated in severe cases. Additionally, antibiotic use in these patients might have contributed to the observed differences in bacterial metabolites like pantothenic acid and indole-3-carboxaldehyde, by altering microbiota composition. While our study provides a comprehensive metabolomic analysis of saliva in COVID-19 patients, future studies should incorporate detailed treatment records to assess their direct impact on metabolic changes. Nevertheless, the robust differences observed between moderate and severe cases indicate that metabolic shifts due to disease progression are key contributors to the observed profiles.

## 5. Conclusions

This study documents strongly significant differences in the metabolome of the saliva of patients infected with SARS-CoV-2 and presenting with moderate COVID-19 disease, particularly in dipeptides and amino acids, probably because of specific proteases. Metabolome differences in severe cases were also highly significant, but with a completely different pattern, where metabolites previously associated with damage to the kidney, heart, brain, and liver were significantly increased. Some of these markers were further increased in deceased patients. This study shows the convenience of saliva for exhaustive analyses of metabolic profiles, particularly in patients with COVID-19, and suggests its potential utility for diagnosis, prognosis, and even pathogenesis studies.

## Figures and Tables

**Table 1:** General characteristics of the clinical groups studied.

Group	Number of Cases	Description
Asymptomatic cases (ACs)	30	Asymptomatic cases, negative for SARS-CoV-2 infection
Ambulatory patients (APs)	102	Ambulatory patients, PCR+ for SARS-CoV-2
Hospitalized patients (HPs)	61	Patients that required hospitalization because of severe symptoms, PCR+ for SARS-CoV-2
Total studied	210	

**Table 2:** Origin of metabolites showing significant association with severity of COVID-19 origin: microbial, environmental, human, or drugs.

	Microbial	Human	Environment	Drugs
**AC vs. AP**	N-Methylisoleucine	N-Acetylserine	Val-Glu	S-Adenosyl-methionine
	Indole-3-carboxaldehyde	N-Methylserine	Ser-Pro-Arg	N,N Diethyl-2-aminoethanol
	Muramic acid	gamma-aminobutyric acid	Serine	DMSO
		Guanidine	Phenylalanine	Isradipine
		Inositol	Tyrosine	
		L-Homocitrulline	Gluconolactone	Xanthine
		N-Acetylhistidine	1,4-Cyclohexanedicarboxylic acid	Mannitol
		Met-Gln	Fluorene	Cefdinir
		Arachidonyl dopamine	Tryptophan	Benzphetamine
		Acetylenedicarboxylic acid	2-Amino-4-tert-butylphenol	
		Uridine diphosphate galactose		
		Glyceraldehyde		
		4-Pyridoxic acid		
**AC vs. HP**	Pantothenic acid	N8-Acetylspermidine	Theobromine	Acetaminophen sulfate
	1-(2-Hydroxyethyl)-2,2,6,6-tetramethyl-4-piperidinol	3-Methylcytidine	Theophylline	Ornidazole
	Indole-3-carboxaldehyde	1,7 Dimethyluric acid	Caffeine	Xanthine
		N-Acetylneuraminic acid	Aconitic acid	Cefdinir
	N-Cinnamoylglycine	Dehydroisoandrosterone sulfate	Diethanolamine	
		Oxypurinol	1,4-Cyclohexanedicarboxylic acid	
		N-Acetylhistidine	Ferulic acid	
		Targinine	Catechol	
		Uracil		
		5,6-Dihydrouracil		
		Uric acid		
**AP vs. HP**	Muramic acid	N1-Acetylspermine	Ser-Pro-Arg	S-Adenosyl-methionine
	2-Amino-1-phenylethanol	8-Oxo-2-deoxyadenosine	Met-Gln	DMSO
	Allantoic acid	N-Methylisoleucine	Val-Glu	Cefdinir
	3-Hydroxyanthranilic acid	Acetylenedicarboxylic acid	Phenylalanine	Benzphetamine
		N8-Acetylspermidine	PyroGlu-Pro	Mannitol
		N-Methylserine	Tyrosine	Methotrexate
		Serine	Phenylacetaldehyde isomer	Isradipine
		Porphobilinogen	Gluconolactone	
		3-Methylcytidine	Theobromine	
		Diethyloxalpropionate	Theophylline	
		N-Acetylserine		
		Uridine diphosphate galactose	Fluorene	
		Cytidine 5′-diphosphocholine	2-Amino-4-tert-butylphenol	
			Pipecolic acid	
			Melamine	
			Valine	

## Data Availability

The original contributions present in this study are included in the article.

## Supplementary Materials

The following supporting information can be downloaded at https://www.mdpi.com/article/10.3390/metabo15030192/s1. Figure S1: A Principal Component Analysis; Figure S2: Volcano plot; Table S1: Raw data; Table S2: Differences in intensity of the selected metabolites by group.

## References

[1] COVID-19 Epidemiological Update—13 February 2025. https://www.who.int/publications/m/item/covid-19-epidemiological-update-edition-176.

[2] Sievers B.L., Cheng M.T.K., Csiba K., Meng B., Gupta R.K. (2024). SARS-CoV-2 and Innate Immunity: The Good, the Bad, and the “Goldilocks”. Cell. Mol. Immunol..

[3] Tang Y., Liu J., Zhang D., Xu Z., Ji J., Wen C. (2020). Cytokine Storm in COVID-19: The Current Evidence and Treatment Strategies. Front. Immunol..

[4] Mollentze N., Streicker D.G. (2020). Viral Zoonotic Risk Is Homogenous among Taxonomic Orders of Mammalian and Avian Reservoir Hosts. Proc. Natl. Acad. Sci. USA.

[5] Dewhirst F.E., Chen T., Izard J., Paster B.J., Tanner A.C.R., Yu W.-H., Lakshmanan A., Wade W.G. (2010). The Human Oral Microbiome. J. Bacteriol..

[6] Tsang T.K., Lee K.H., Foxman B., Balmaseda A., Gresh L., Sanchez N., Ojeda S., Lopez R., Yang Y., Kuan G. (2020). Association Between the Respiratory Microbiome and Susceptibility to Influenza Virus Infection. Clin. Infect. Dis..

[7] Larios Serrato V., Meza B., Gonzalez-Torres C., Gaytan-Cervantes J., González Ibarra J., Santacruz Tinoco C.E., Anguiano Hernández Y.-M., Martínez Miguel B., Cázarez Cortazar A., Sarquiz Martínez B. (2023). Diversity, Composition, and Networking of Saliva Microbiota Distinguish the Severity of COVID-19 Episodes as Revealed by an Analysis of 16S rRNA Variable V1-V3 Region Sequences. mSystems.

[8] Moreno E., Ciordia S., Fátima S.M., Jiménez D., Martínez-Sanz J., Vizcarra P., Ron R., Sánchez-Conde M., Bargiela R., Sanchez-Carrillo S. (2024). Proteomic Snapshot of Saliva Samples Predicts New Pathways Implicated in SARS-CoV-2 Pathogenesis. Clin. Proteom..

[9] Zhang C.-Z., Cheng X.-Q., Li J.-Y., Zhang P., Yi P., Xu X., Zhou X.-D. (2016). Saliva in the Diagnosis of Diseases. Int. J. Oral. Sci..

[10] Blaženović I., Kind T., Sa M.R., Ji J., Vaniya A., Wancewicz B., Roberts B.S., Torbašinović H., Lee T., Mehta S.S. (2019). Structure Annotation of All Mass Spectra in Untargeted Metabolomics. Anal. Chem..

[11] Tsugawa H., Cajka T., Kind T., Ma Y., Higgins B., Ikeda K., Kanazawa M., VanderGheynst J., Fiehn O., Arita M. (2015). MS-DIAL: Data-Independent MS/MS Deconvolution for Comprehensive Metabolome Analysis. Nat. Methods.

[12] Fan S., Kind T., Cajka T., Hazen S.L., Tang W.H.W., Kaddurah-Daouk R., Irvin M.R., Arnett D.K., Barupal D.K., Fiehn O. (2019). Systematic Error Removal Using Random Forest for Normalizing Large-Scale Untargeted Lipidomics Data. Anal. Chem..

[13] Wickham H. (2016). Ggplot2: Elegant Graphics for Data Analysis.

[14] Love M.I., Huber W., Anders S. (2014). Moderated Estimation of Fold Change and Dispersion for RNA-Seq Data with DESeq2. Genome Biol..

[15] Ansone L., Briviba M., Silamikelis I., Terentjeva A., Perkons I., Birzniece L., Rovite V., Rozentale B., Viksna L., Kolesova O. (2021). Amino Acid Metabolism Is Significantly Altered at the Time of Admission in Hospital for Severe COVID-19 Patients: Findings from Longitudinal Targeted Metabolomics Analysis. Microbiol. Spectr..

[16] Bourgin M., Derosa L., Silva C.A.C., Goubet A.-G., Dubuisson A., Danlos F.-X., Grajeda-Iglesias C., Cerbone L., Geraud A., Laparra A. (2021). Circulating Acetylated Polyamines Correlate with Covid-19 Severity in Cancer Patients. Aging.

[17] Frampas C.F., Longman K., Spick M.P., Lewis H.M., Costa C.D.S., Stewart A., Dunn-Walters D., Greener D., Evetts G.E., Skene D. (2021). Untargeted Saliva Metabolomics Reveals COVID-19 Severity: Saliva Metabolomics for SARS-CoV-2 Prognosis. bioRxiv.

[18] Pozzi C., Levi R., Braga D., Carli F., Darwich A., Spadoni I., Oresta B., Dioguardi C.C., Peano C., Ubaldi L. (2022). A “Multiomic” Approach of Saliva Metabolomics, Microbiota, and Serum Biomarkers to Assess the Need of Hospitalization in Coronavirus Disease 2019. Gastro Hep Adv..

[19] Costa Dos Santos Junior G., Pereira C.M., Kelly da Silva Fidalgo T., Valente A.P. (2020). Saliva NMR-Based Metabolomics in the War Against COVID-19. Anal. Chem..

[20] Thomadaki K., Helmerhorst E.J., Tian N., Sun X., Siqueira W.L., Walt D.R., Oppenheim F.G. (2011). Whole-Saliva Proteolysis and Its Impact on Salivary Diagnostics. J. Dent. Res..

[21] Garreto L., Charneau S., Mandacaru S.C., Nóbrega O.T., Motta F.N., de Araújo C.N., Tonet A.C., Modesto F.M.B., Paula L.M., de Sousa M.V. (2021). Mapping Salivary Proteases in Sjögren’s Syndrome Patients Reveals Overexpression of Dipeptidyl Peptidase-4/CD26. Front. Immunol..

[22] Turk B. (2006). Targeting Proteases: Successes, Failures and Future Prospects. Nat. Rev. Drug Discov..

[23] Human Metabolome Database: Showing Metabocard for Valylglutamine (HMDB0029125). https://hmdb.ca/metabolites/HMDB0029125.

[24] Minen R.I., Thirumalaikumar V.P., Skirycz A. (2023). Proteinogenic Dipeptides, an Emerging Class of Small-Molecule Regulators. Curr. Opin. Plant Biol..

[25] Roager H.M., Licht T.R. (2018). Microbial Tryptophan Catabolites in Health and Disease. Nat. Commun..

[26] Frampas C.F., Longman K., Spick M., Lewis H.-M., Costa C.D.S., Stewart A., Dunn-Walters D., Greener D., Evetts G., Skene D.J. (2022). Untargeted Saliva Metabolomics by Liquid Chromatography-Mass Spectrometry Reveals Markers of COVID-19 Severity. PLoS ONE.

[27] Wen D., Zheng Z., Surapaneni A., Yu B., Zhou L., Zhou W., Xie D., Shou H., Avila-Pacheco J., Kalim S. (2022). Metabolite Profiling of CKD Progression in the Chronic Renal Insufficiency Cohort Study. JCI Insight.

[28] Luo S., Surapaneni A., Zheng Z., Rhee E.P., Coresh J., Hung A.M., Nadkarni G.N., Yu B., Boerwinkle E., Tin A. (2020). Variants, N-Acetylated Amino Acids, and Progression of CKD. Clin. J. Am. Soc. Nephrol..

[29] Goetzman E., Gong Z., Rajasundaram D., Muzumdar I., Goodchild T., Lefer D., Muzumdar R. (2022). Serum Metabolomics Reveals Distinct Profiles during Ischemia and Reperfusion in a Porcine Model of Myocardial Ischemia-Reperfusion. Int. J. Mol. Sci..

[30] Hammond T.C., Xing X., Yanckello L.M., Stromberg A., Chang Y.-H., Nelson P.T., Lin A.-L. (2021). Human Gray and White Matter Metabolomics to Differentiate APOE and Stage Dependent Changes in Alzheimer’s Disease. J. Cell. Immunol..

[31] Ree R., Varland S., Arnesen T. (2018). Spotlight on Protein N-Terminal Acetylation. Exp. Mol. Med..

[32] Zelante T., Iannitti R.G., Cunha C., De Luca A., Giovannini G., Pieraccini G., Zecchi R., D’Angelo C., Massi-Benedetti C., Fallarino F. (2013). Tryptophan Catabolites from Microbiota Engage Aryl Hydrocarbon Receptor and Balance Mucosal Reactivity via Interleukin-22. Immunity.

[33] Strickland W.N., Strickland M., de Groot P.C., Von Holt C., Wittmann-Liebold B. (1980). The Primary Structure of Histone H1 from Sperm of the Sea Urchin Parechinus Angulosus. 1. Chemical and Enzymatic Fragmentation of the Protein and the Sequence of Amino Acids in the Four N-Terminal Cyanogen Bromide Peptides. Eur. J. Biochem..

[34] Pascale R.M., Simile M.M., Calvisi D.F., Feo C.F., Feo F. (2022). S-Adenosylmethionine: From the Discovery of Its Inhibition of Tumorigenesis to Its Use as a Therapeutic Agent. Cells.

[35] Aboud O., Liu Y., Dahabiyeh L., Abuaisheh A., Li F., Aboubechara J.P., Riess J., Bloch O., Hodeify R., Tagkopoulos I. (2023). Profile Characterization of Biogenic Amines in Glioblastoma Patients Undergoing Standard-of-Care Treatment. Biomedicines.

[36] Magnúsdóttir S., Ravcheev D., de Crécy-Lagard V., Thiele I. (2015). Systematic Genome Assessment of B-Vitamin Biosynthesis Suggests Co-Operation among Gut Microbes. Front. Genet..

[37] Lee J.-H., Lee J. (2010). Indole as an Intercellular Signal in Microbial Communities. FEMS Microbiol. Rev..

[38] Wallace H.M., Fraser A.V., Hughes A. (2003). A Perspective of Polyamine Metabolism. Biochem. J..

[39] Nayak A., Liu C., Mehta A., Ko Y.-A., Tahhan A.S., Dhindsa D.S., Uppal K., Jones D.P., Butler J., Morris A.A. (2020). N8-Acetylspermidine: A Polyamine Biomarker in Ischemic Cardiomyopathy With Reduced Ejection Fraction. J. Am. Heart Assoc..

[40] Qu Y., Eguchi A., Ma L., Wan X., Mori C., Hashimoto K. (2023). Role of the Gut-Brain Axis via the Subdiaphragmatic Vagus Nerve in Stress Resilience of 3,4-Methylenedioxymethamphetamine in Mice Exposed to Chronic Restrain Stress. Neurobiol. Dis..

[41] Wenzel A.G., Reiner J.L., Kohno S., Wolf B.J., Brock J.W., Cruze L., Newman R.B., Kucklick J.R. (2021). Biomonitoring of Emerging DINCH Metabolites in Pregnant Women in Charleston, SC: 2011–2014. Chemosphere.

[42] Bohnsack K.E., Kleiber N., Lemus-Diaz N., Bohnsack M.T. (2022). Roles and Dynamics of 3-Methylcytidine in Cellular RNAs. Trends Biochem. Sci..

[43] Aarsand A.K., Petersen P.H., Sandberg S. (2006). Estimation and Application of Biological Variation of Urinary Delta-Aminolevulinic Acid and Porphobilinogen in Healthy Individuals and in Patients with Acute Intermittent Porphyria. Clin. Chem..

