# Supplementary material for: Analyses of Saliva Metabolome Reveal Patterns of Metabolites That Differentiate SARS-CoV-2 Infection and COVID-19 Disease Severity
Source: Metabolites. 2025 Mar 11;15(3):192. doi: 10.3390/metabo15030192 (PMC11944064; doi:10.3390/metabo15030192)
Supplement: Supplementary file 1 [file metabolites-15-00192-s001.zip › Supplemental Figure S1.jpg.pdf]

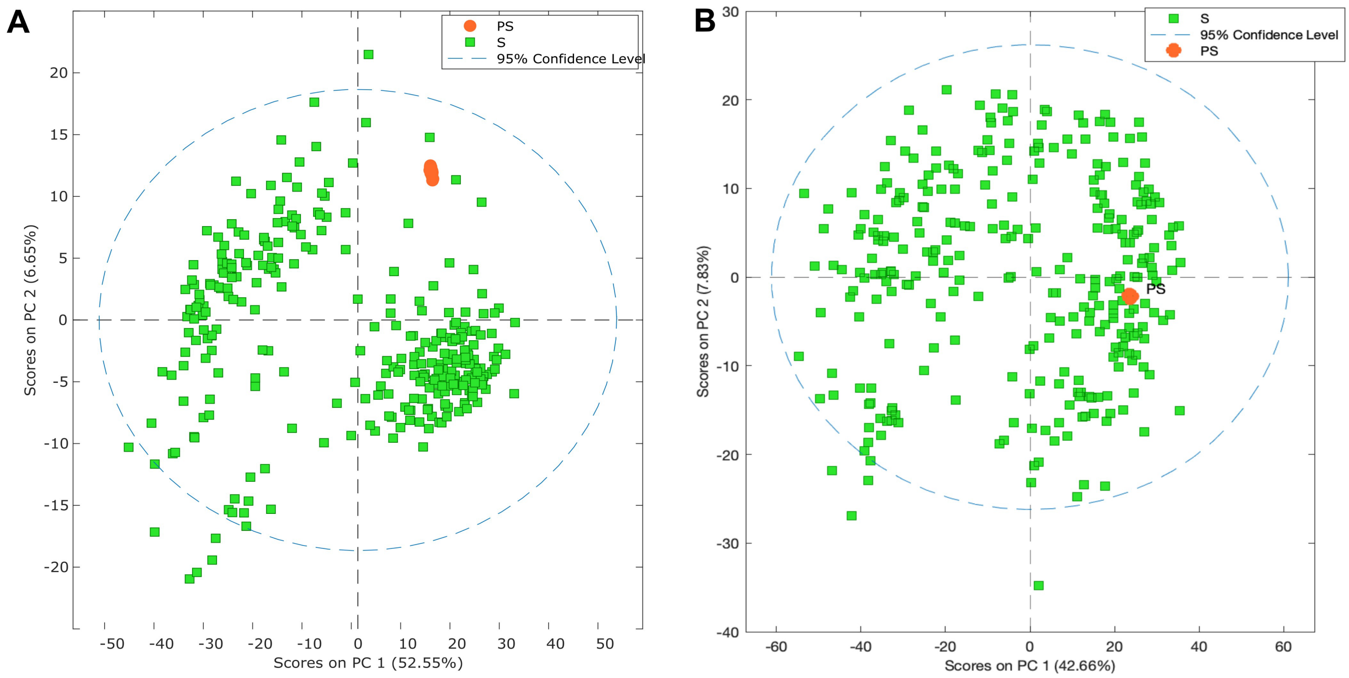

Supplemental Figure S1. A Principal Component Analysis (PCA). A) positive, and B) negative data matrices (308 x 2218 and 308 x 1234).
