# Supplementary figures and images for: Analyses of Saliva Metabolome Reveal Patterns of Metabolites That Differentiate SARS-CoV-2 Infection and COVID-19 Disease Severity
Source: Metabolites. 2025 Mar 11;15(3):192. doi: 10.3390/metabo15030192 (PMC11944064; doi:10.3390/metabo15030192)

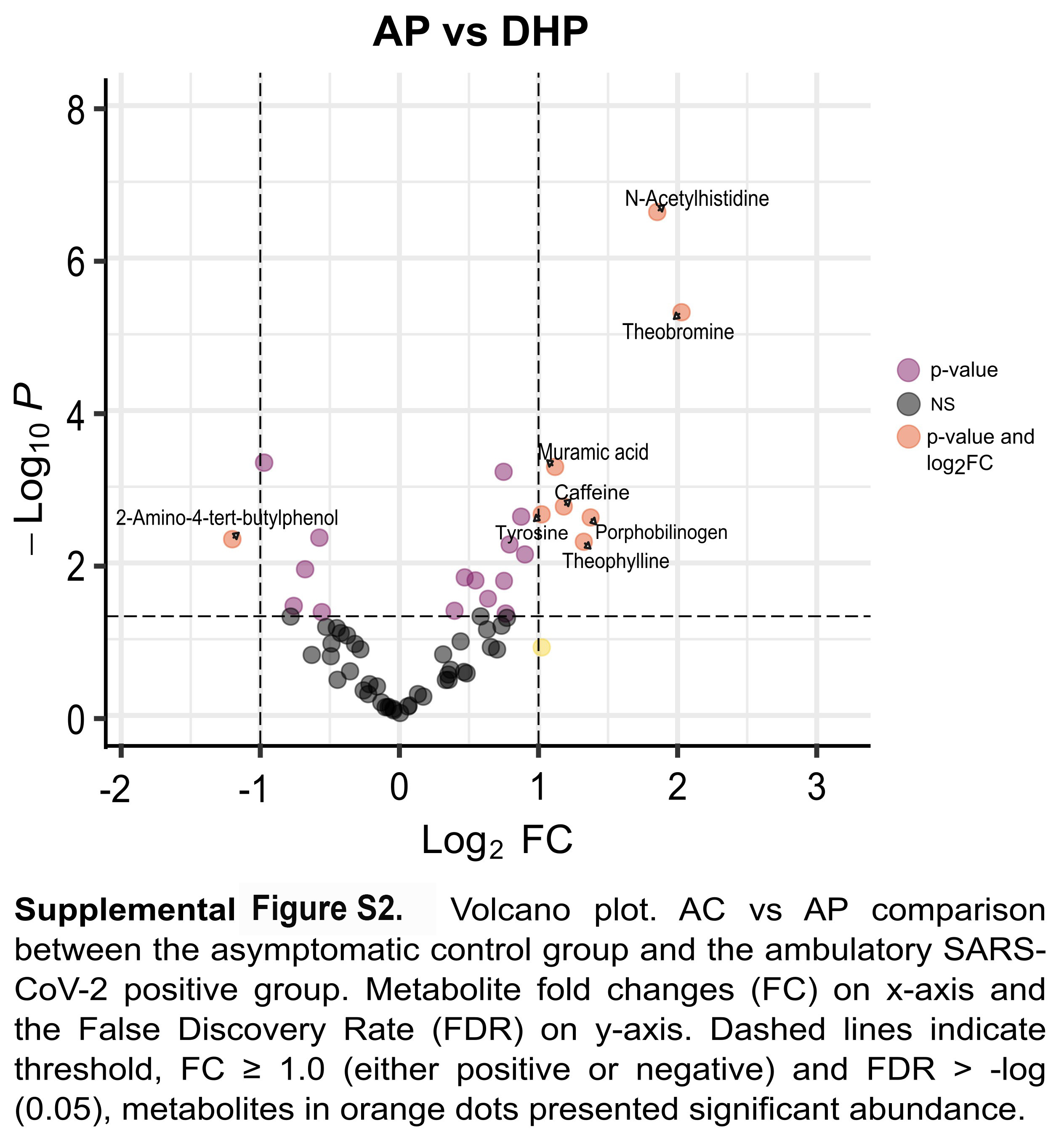

Supplement: Supplementary file 1 [file metabolites-15-00192-s001.zip › Supplemental Figure S2.jpg]
